# Supplementary material for: Effects of Environmental Factors on the Spatial Distribution Pattern and Diversity of Insect Communities along Altitude Gradients in Guandi Mountain, China
Source: Insects. 2023 Feb 24;14(3):224. doi: 10.3390/insects14030224 (PMC10058187; doi:10.3390/insects14030224)
Supplement: Supplementary file 1 [file insects-14-00224-s001.zip › insects-2187387-supplementary.pdf]

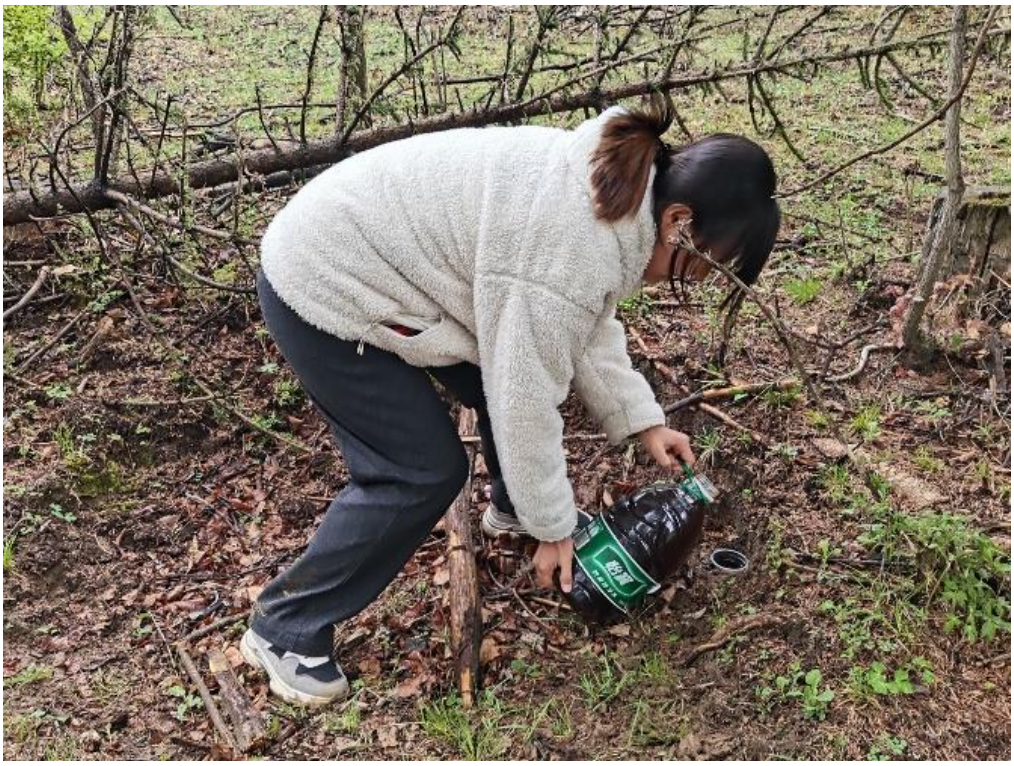

Figure S1 Field operation of "pitfall trapping".

Table S1. The geographical coordinates of seven typical vegetation community ecosystems along the altitude gradient in the Guandi Mountain.

Vegetation community	Elevation (m)	Longitude	Latitude
		37°44′00″	111°30′15″
QWF	1600	37°49′31″	111°28′27″
		37°49′01″	111°28′35″
		37°49′33″	111°29′42″
PTF	1800	37°49′34″	111°29′43″
		37°49′16″	111°28′25″
		37°49′29″	111°27′11″
PBM	2000	37°52′34″	111°26′21″
		37°53′13″	111°25′53″
		37°52′18″	111°26′47″
PWF	2200	37°52′33″	111°26′22″
		37°53′81″	111°25′44″
		37°53′63″	111°25′40″
PLF	2400	37°51′36″	111°33′22″
		37°51′49″	111°33′13″
		37°51′34″	111°33′41″
LPF	2600	37°52′21″	111°33′19″
		37°52′44″	111°32′56″
		37°52′40″	111°33′10″
SAM	2800	37°52′32″	111°33′22″
		37°44′02″	111°30′15″

Table S2 Analysis of physicochemical properties of 0-10 cm soil layer in typical vegetation community ecosystems along altitudinal gradient of the Guandi Mountains

Elevation (m)	Temperature (℃)	Relative humidity (%)	pH	Electrical conductivity (us/cm)	Available N (mg/kg)	Available P (mg/kg)	Available K (mg/kg)	N/P	N/K
1600	18.99±1.15 [a] a	18.56±4.77 bc	5.81±0.57 b	138.80±18.18 ab	12.27±1.39 ab	16.53±1.77 ab	41.80±4.43 ab	0.74±0.013 a	0.29±0.009 a
1800	17.13±0.53 b	16.37±6.26 c	5.99±0.61 b	137.13±15.20 abc	12.07±1.28 abc	16.33±1.68 ab	41.47±4.29 ab	0.74±0.011 a	0.29±0.005 a
2000	16.26±2.63 b	24.73±9.13 a	5.91±0.37 b	135.27±8.09 abc	11.87±0.83 abc	16.07±1.10 bc	40.60±2.29 bc	0.74±0.025 a	0.29±0.012 a
2200	13.50±0.60 c	14.95±3.87 c	6.49±0.27 a	128.87±3.85 c	11.20±0.41 c	15.27±0.59 c	39.07±1.39 c	0.74±0.028 a	0.29±0.011 a
2400	13.25±0.95 c	18.28±5.37 c	6.31±0.21 a	128.80±3.73 c	11.40±0.51 c	15.40±0.51 c	38.93±1.03 c	0.74±0.017 a	0.29±0.010 a
2600	13.62±0.94 c	22.95±5.48 ab	5.97±0.30 b	131.93±4.35 bc	11.67±0.49 bc	15.73±0.59 bc	39.93±1.53 bc	0.74±0.012 a	0.29±0.006 a
2800	19.00±1.72 a	26.76±1.83 a	5.07±0.17 c	142.22±6.38 a	12.67±0.50 a	17.02±0.50 a	43.11±1.36 a	0.75±0.026 a	0.29±0.009 a
F-value	46.343***	7.588***	14.054***	2.412*	3.281**	3.921**	3.815**	0.155	0.414
P-value	$1.01*10^{-24}$	$1.25*10^{-6}$	$2.7*10^{-11}$	0.043	0.006	0.002	0.002	0.988	0.868

[a] Values are mean±SD. For each column, values with different letters are significantly different at $P = 0.05$.

Table S2 Analysis of physicochemical properties of 0-10 cm soil layer in typical vegetation community ecosystems along altitudinal gradient of the Guandi Mountains (continued)

Elevation (m)	P/K	Bulk density (g/cm^3)	Maximum water holding capacity (%)	Capillary water holding capacity (%)	Noncapillary porosity (%)	Capillary porosity (%)	Total soil porosity (%)
1600	0.40±0.009 [b] a	1.02±0.04 b	50.25±5.53 a	46.23±4.58 a	3.94±1.97 ab	45.40±5.88 a	49.34±6.74 a
1800	0.39±0.005 a	1.08±0.10 ab	45.93±6.56 ab	43.89±6.83 ab	1.91±0.80 c	41.21±10.09 a	43.12±10.16 a
2000	0.40±0.007 a	1.01±0.07 b	42.41±5.61 bc	38.10±5.28 bc	4.31±1.13 ab	38.09±6.04 a	42.40±6.50 a
2200	0.39±0.008 a	1.20±0.11 a	34.54±8.31 d	30.62±7.87 d	3.31±1.14 bc	25.39±5.97 b	28.70±6.51 b
2400	0.40±0.008 a	1.23±0.23 a	32.92±4.41 d	28.58±5.24 d	3.85±2.11 ab	23.44±2.87 b	27.28±3.92 b
2600	0.39±0.008 a	1.03±0.08 b	35.28±5.03 d	30.76±5.09 d	5.33±1.20 ab	30.13±5.89 b	34.42±4.80 b
2800	0.39±0.005 a	1.17±0.05 a	38.78±2.23 cd	35.00±3.04 cd	5.67±1.82 d	29.84±2.86 b	33.04±1.77 b
F-value	0.67	3.966**	7.921***	3.966**	7.921***	9.113***	3.997**
P-value	0.674	0.004	2.20*10-5	0.004	2.20*10-5	5.83*10-6	0.004

[a] Values are mean±SD. For each column, values with different letters are significantly different at P = 0.05.

Table S3. List of the collected insects in the Guandi Mountain.

Order	Family	Species	Number of individuals
Coleoptera	Alticinae	*Altica cirsicola* (Ohno,1960)	1
Coleoptera	Alticinae	*Altica weisei* Jacobson	16
Coleoptera	Attelabidae	*Apoderus coryli* Linnaeus	6
Coleoptera	Buprestidae	*Scintillatrix bellula*	2
Coleoptera	Buprestidae	*Paracylindromorphus japanensis* (Saunders,1873)	1
Coleoptera	Carabidae	*Apotomopterus maacki maacki* (Morawitz)	13
Coleoptera	Carabidae	*Calosoma chinense* Kirby	9
Coleoptera	Carabidae	*Anisodactylus signatus* (Panzer)	108
Coleoptera	Carabidae	*Harpalus griseus* (Panzer)	13
Coleoptera	Carabidae	*Harpalus variipes* Bates	16
Coleoptera	Carabidae	*Hololius ceylanicus* (Nietner,1856)	10
Coleoptera	Carabidae	*Carabus (Ctenocarabus) melancholicus* (Fabricius,1798)	217
Coleoptera	Carabidae	*Carabus (Procrustes) anatolicus*	139
Coleoptera	Carabidae	*Carabus (Rhigocarabus) korsakowi*	267
Coleoptera	Carabidae	*Carabus brandti* Faldermann	29
Coleoptera	Carabidae	*Carbus vladimirskyi* Dejean	3
Coleoptera	Carabidae	*Diplocheila zeelandica* Redtenbacher,1867	193
Coleoptera	Carabidae	*Dolichus halensis* (Schaller)	7
Coleoptera	Carabidae	*Harpalus sinicus* Hope,1845	27
Coleoptera	Carabidae	*Leptocarabus kurilensis* (Lapoug)	57
Coleoptera	Carabidae	*Nebria livida* Linnaeus	66
Coleoptera	Carabidae	*Scaphinotus angusticollis* (Mannerheim 1823)	1
Coleoptera	Cerambycidae	*Anoplophora glabripennis* (Motschulsky,1854)	6
Coleoptera	Cerambycidae	*Aromia bungii* (Faldermann)	1
Coleoptera	Cerambycidae	*Dorysthenes paradoxus* (Faldermann,1833)	5

Coleoptera	Chrysomelidae	*Chrysolina aurichalcea* (Mannerheim,1825)	1
Coleoptera	Chrysomelidae	*Chrysolina exanthematica* (Wiedemann,1821)	1
Coleoptera	Chrysomelidae	*Chrysolina aeruginosa* Fa1d	19
Coleoptera	Chrysomelidae	*Chrysolina populi* Linnaeus	5
Coleoptera	Chrysomelidae	*Entomoscelis orientalis* Mots	48
Coleoptera	Chrysomelidae	*Monolepta hieroglyphica* (Motschulsky)	13
Coleoptera	Chrysomelidae	*Pallasiola absinthii* (Pallas)	2
Coleoptera	Chrysomelidae	*Phratora vitellinae* (Linnaeus,1758)	1
Coleoptera	Cicindelidae	*Cicindela hybrida transbaicalica* Motschulky	2
Coleoptera	Coccinellidae	*Aiolocariamirabilis* (Motschulsky,1860)	6
Coleoptera	Coccinellidae	*Coccinella septempunctata* (Linnaeus)	3
Coleoptera	Coccinellidae	*Anatis ocellata* (Linnaeus)	20
Coleoptera	Coccinellidae	*Coccinulla quatuordecimpustulata* (Linnaeus,1758)	65
Coleoptera	Coccinellidae	*Harmonia axyridis* Pallas	29
Coleoptera	Coccinellidae	*Hippodamia tredecimpunctata* (Linnaeus)	3
Coleoptera	Coccinellidae	*Lemnia saucia* (Mulsant)	1
Coleoptera	Coccinellidae	*Propylaea japonica* (Thunberg,1781)	6
Coleoptera	Curculionidae	*Chlorophanus auripes* Faust,1897	60
Coleoptera	Curculionidae	*Cyllorhynchites ursulus* (Roelofs)	3
Coleoptera	Curculionidae	*Hylobius abietis haroldii*	1
Coleoptera	Elateridae	*Agriotes subvittatus* Motschulsky	4
Coleoptera	Elateridae	*Agrypnus argillaceus* (Solsky)	1
Coleoptera	Elateridae	*Melanotus caudex* Lewis	51
Coleoptera	Elateridae	*Selatosomus latus* (Fabricius,1801)	2
Coleoptera	Eumolpidae	*Colasposoma dauricum* Motschulsky	9
Coleoptera	Galerucinae	*Agelastica alni* (Linnaeus,1758)	9

Coleoptera	Halticidae	*Phyllotreta striolata* (Fabricius)	2
Coleoptera	Halticidae	*Hemipyxis plagioderoides* (Motschulsky)	11
Coleoptera	Halticidae	*Psylliodes attenuata* Koch	4
Coleoptera	Halticidae	*Psylliodes reitteri* Weise	28
Coleoptera	Meloidae	*Epicauta chinensis* Laporte,1840	1
Coleoptera	Meloidae	*Epicauta hirticornis* (Haag-Rutenburg,1880)	9
Coleoptera	Nitidulidae	*Carpophilus delkeskampi* (Hisamatsu)	1
Coleoptera	Nitidulidae	*Carpophilus nitidus* Miurray	1
Coleoptera	Rutelidae	*Mimela holosericea* (Fabricius,1787)	8
Coleoptera	Scarabaeidae	*Aphodius nigrotessellatus* Nakane,1972	12
Coleoptera	Scarabaeidae	*Onthophagus fodiens* Waterhouse,1875	2
Coleoptera	Scarabaeidae	*Catharsius molossus* Linnaeus	6
Coleoptera	Scolytidae	*Dendroctonus valens* LeConte ,1859	1
Coleoptera	Scolytidae	*Phloeosinus aubei* (Perris,1855)	2
Coleoptera	Scolytidae	*Pityogenes chalcographus* Bedel,1888	35
Coleoptera	Silphidae	*Nicrophorus concolor* Kraatz,1877	4
Coleoptera	Silphidae	*Nicrophorus vespilloides* Herbst,1783	2
Coleoptera	Staphylinidae	*Bolitochara lucida* (Gravenhorst,1802)	109
Coleoptera	Staphylinidae	*Leptacinus batychrus* Gyllenhal	6
Coleoptera	Staphylinidae	*Ontholestes gracilis* Sharp,1874	3
Coleoptera	Staphylinidae	*Pinophilus punctatissimus* Sharp,1889	292
Coleoptera	Tenebrionidae	*Blaps chinensis* Faldermann	1
Diptera	Asilidae	*Erax laleralis*	18

Diptera	Bombyliidae	*Anthrax aygulus* Fabricius,1805	2
Diptera	Bombyliidae	*Bombylius major* Linnaeus,1758	18
Diptera	Bombyliidae	*Bombylius beijingensis*	2
Diptera	Calliphoridae	*Chrysomyia megacephala* (Fabricius,1794)	59
Diptera	Calliphoridae	*Lucilia illustri* (Meigen,1826)	16
Diptera	Calliphoridae	*Calliphora vicina* Robineau-Desvoidy,1830	38
Diptera	Calliphoridae	*Lucilia sericata* (Meigen,1826)	6
Diptera	Cecidomyiidae	*Mayetiola destructor* (Say,1817)	2
Diptera	Cecidomyiidae	*Rhopalomyia californica* Felt,1908	4
Diptera	Culicidae	*Anopheles sinensis* Wiedemann,1828	4
Diptera	Culicidae	*Anopheles lindesayi* Giles,1900	6
Diptera	Culicidae	*Culex pipiens pallens* Coquillett,1898	88
Diptera	Drosophilidae	*Drosophila melanogaster* Meigen,1830	70
Diptera	Drosophilidae	*Drosophila (Drosophila) hydei* Strtevant	6
Diptera	Ephydridae	*Hydrellia griseola* (Fallen)	37
Diptera	Muscidae	*Hebecnema affinis* Malloch,1921	60
Diptera	Muscidae	*Musca domestica* Linnaeus,1758	561
Diptera	Muscidae	*Hydrotaea* sp.	299
Diptera	Sarcophagidae	*Helicophagella melanura*	34
Diptera	Sarcophagidae	*Boettcherisca peregrina* (Robineau-Desvoidy,1830)	7
Diptera	Scathophagidae	*Scathophaga stercoraria* (Linnaeus,1758)	311
Diptera	Stratiomyidae	*Hermetia illucens* L.	3
Diptera	Syrphidae	*Chrysotoxum sp.*	6
Diptera	Syrphidae	*Eristalis cerealis* Fabricius,1805	3
Diptera	Syrphidae	*Metasyrphus nitens* (Zetterstedt,1843)	9

Diptera	Syrphidae	*Sphaerophoria indiana* Bigot,1884	162
Diptera	Syrphidae	*Episyrphus balteatus* (Geer,1776)	21
Diptera	Tabanidae	*Haematopota pekingensis* Linnaeus	32
Diptera	Tachinidae	*Prosena siberita* (Fabricius,1775)	30
Diptera	Tipulidae	*Nephrotoma shanxiensis* Yang and Yang,1990	34
Diptera	Tipulidae	*Nephrotoma* sp.	1
Diptera	Tipulidae	*Tipula (Yamatotipula) anio* Alexander	4
Diptera	Tipulidae	*Nephrotoma aculeata* (Loew,1871)	40
Diptera	Tipulidae	*Tipula nova* Walker	75
Orthoptera	Acrididae	*Acrida cinerea* (Thunberg,1815)	7
Orthoptera	Acrididae	*Mongolotettix japonicus vittatus* (Uvarov,1914)	1
Orthoptera	Arcypteridae	*Chorthippus brunneus* (Thunberg,1815)	1
Orthoptera	Arcypteridae	*Chorthippus fallax* (Zubovski,1900)	2
Orthoptera	Arcypteridae	*Euchorthippus unicolor* (Ikonnikov,1913)	77
Orthoptera	Arcypteridae	*Omocestus haemorrhoidalis* (Charpentier,1825)	2
Orthoptera	Catantopidae	*Calliptamus abbreviatus* Ikonnikov,1913	11
Orthoptera	Catantopidae	*Oxya chinensis* (Thunberg)	278
Orthoptera	Catantopidae	*Shirakiacris shirakii* (Bolívar,I.,1914)	1
Orthoptera	Cedipodidae	*Gastrimargus marmoratus* (Thunberg,1815)	1
Orthoptera	Cedipodidae	*Celes akitanus* (Shiraki,1910)	1
Orthoptera	Gomphoceridae	*Myrmeleotettix longipennis* Zhang, 1984	31
Orthoptera	Gryllidae	*Brachytrupes portentosus* (Lichtenstein,1796)	15
Orthoptera	Gryllidae	*Loxoblemmus doenitzi* Stein,1881	4

Orthoptera	Gryllidae	*Teleogryllus infernalis* (Saussure,1877)	2
Orthoptera	Gryllotalpidae	*Gryllotalpa orientalis* Burmeister,1838	68
Orthoptera	Gryllotalpidae	*Gryllotalpa unispina* Saussure,1874	9
Orthoptera	Oedipodidae	*Angaracris rhodopa* (Fischer, Waldheim,1846)	1
Orthoptera	Oedipodidae	*Bryodemella tuberculatum dilutum* (Stoll, 1813)	1
Orthoptera	Oedipodidae	*Epacromius coerulipes* (Ivanov,1888)	1
Orthoptera	Oedipodidae	*Locusta migratoria manilensis* (Meyen,1835)	35
Orthoptera	Oedipodidae	*Oedaleus asiaticus* Bei-Bienko,1941	1
Orthoptera	Oedipodidae	*Oedaleus infernalis* Saussure,1884	198
Orthoptera	Oedipodidae	*Sphingonotus mongolicus* Saussure,1888	2
Orthoptera	Oedipodidae	*Trilophidia annulata* (Thunberg,1815)	5
Orthoptera	Pamphagidae	*Haplotropis brunneriana* Saussure,1888	281
Orthoptera	Pyrgomorphidae	*Atractomorpha lata* (Mochulsky,1866)	8
Orthoptera	Pyrgomorphidae	*Atractomorpha sinensis* Bolívar,I.,1905	3
Orthoptera	Pyrgomorphidae	*Patanga japonica* (Bolívar,I.,1898)	6
Orthoptera	Tetrigidae	*Paratettix uvarovi* Semenov,1915	1
Orthoptera	Tetrigidae	*Tetrix japonica* (Bolívar,I.,1887)	133
Orthoptera	Tettigonidae	*Conocephalus chinensis* (Redtenbacher,1891)	2
Orthoptera	Tettigonidae	*Tettigonia viridissima* (Linnaeus,1758)	17
Orthoptera	Tettigonidae	*Gampsocleis buergeri* (Haan,1842)	4
Orthoptera	Tridactylidae	*Xya japonica* (Haan,1844)	4
Hemiptera	Scutelleridae	*Eurygaster testudinaria* (geoffroy,1785)	30

Hemiptera	Reduviidae	*Cnizocoris sinensis* Kormilev,1957	9
Hemiptera	Cercopidae	*Aphrophora intermedia* Uhler,1896	27
Hemiptera	Cercopidae	*Callitettix versicolor* (Fabricius)	3
Hemiptera	Cicadellidea	*Cicadella viridis* (Linnaeus,1758)	171
Hemiptera	Cicadellidea	*Nephotettix cincticeps* Uhler 1896	232
Hemiptera	Cicadidae	*Oncotympana maculaticollis* (De Motschulsky,1866)	13
Hemiptera	Coreidae	*Chorosoma brevicolle* Hsiao,1964	30
Hemiptera	Coreidae	*Rhopalus latus* (Jakovlev,1883)	11
Hemiptera	Coreidae	*Derepteryx fuliginosa* (Uhler,1860)	6
Hemiptera	Coreidae	*Leptocorisa chinensis* (Fabricius,1775)	17
Hemiptera	Coreidae	*Riptortus pedestris* (Fabricius, 1775)	2
Hemiptera	Dictyoharidae	*Dictyophara patruelis* Stål,1859	9
Hemiptera	Issidae	*Dentatissus damnosus* (Chou & Lu ,1985)	6
Hemiptera	Lygaeidae	*Geocoris sp.*	3
Hemiptera	Lygaeidae	*Panaorus albomaculatus* (Scott,1874)	45
Hemiptera	Lygaeidae	*Lygaeus murinus* (Kiritshenko,1914)	3
Hemiptera	Lygaeidae	*Nysius ericae* (Schilling,1829)	65
Hemiptera	Lygaeidae	*Rhyparochromus (Panaoras) albomaculatus* (Scott,1874)	2
Hemiptera	Membracidae	*Gargara genistae* Fabricius	3
Hemiptera	Membracidae	*Machaerotypus mali* Chou & Yuan 1981	5
Hemiptera	Pentatomidae	*Eurydema gebleri* Kolenati,1846	44
Hemiptera	Pentatomidae	*Halyomorpha halys* (Stål,1855)	23
Hemiptera	Pentatomidae	*Palomena viridissima* (Poda,1761)	9

Hemiptera	Pentatomidae	*Pentatoma semiannulata* (Motschulsky,1860)	12
Hemiptera	Pentatomidae	*Rubiconia intermedia* (Wolff,1811)	137
Hemiptera	Pentatomidae	*Menida scotti* Puton	4
Hemiptera	Pentatomidae	*Plautia fimbriata* (Fabricius,1787)	2
Hemiptera	Pentatomidae	*Stollia ventralis* (Westwood,1837)	26
Hemiptera	Pentatomidae	*Eysarcoris ventralis* (Westwood,1837)	90
Hemiptera	Pentatomidae	*Arma chinensis* (Fallou,1794)	16
Hemiptera	Plataspidae	*Megacopta cribraria* (Fabricius,1798)	3
Hemiptera	Reduviidae	*Coranus lativentris* Jakovlev	6
Hymenoptera	Apidae	*Amegilla parhypate* Lieftinck,1975	15
Hymenoptera	Apidae	*Apis cerana* Fabricius,1793	11
Hymenoptera	Apidae	*Apis mellifera* Linnaeus,1758	6
Hymenoptera	Apidae	*Bombus remotus* (Tkalcu,1968)	18
Hymenoptera	Colletidae	*Colletes gigas* Cockerell,1918	1
Hymenoptera	Formicidae	*Camponotus japonicus* Mayr,1866	372
Hymenoptera	Formicidae	*Formica sinensis* Wheeler,1913	209
Hymenoptera	Formicidae	*Formica neorufibarbis* Emery,1893	35
Hymenoptera	Formicidae	*Formica sinensis* Wheeler, 1913	825
Hymenoptera	Formicidae	*Monomorium pharaonic* (Linnaeus,1758)	291
Hymenoptera	Formicidae	*Tetramorium caespitum* (Linnaeus,1758)	698
Hymenoptera	Formicidae	*Formica cunicularia* Latreille,1798	1
Hymenoptera	Ichneumonidae	*Enicospilus melanocorpus* Cameron	2
Hymenoptera	Ichneumonidae	*Campoletis chlorideae* Uchida,1957	25
Hymenoptera	Ichneumonidae	*Charops bicolor* (Szépligeti,1906)	1

Hymenoptera	Ichneumonidae	*Sphecodes pieli* Cockerell,1931	2
Hymenoptera	Megachilidae	*Megachile remota* Smith	12
Hymenoptera	Scoliidae	*Campsomeris grossa*	2
Hymenoptera	Vespidae	*Vespa mandarinia* (Fabricius,1787)	46
Hymenoptera	Vespidae	*Rhynchium quinquecinctum* (Fabricius)	9
Hymenoptera	Vespidae	*Vespa crabro* Linnaeus,1758	75
Lepidoptera	Hesperiidae	*Daimio tethys* Ménétriés 1857	3
Lepidoptera	Hesperiidae	*Lobocla bifasciata* Bremer & Grey	3
Lepidoptera	Hesperiidae	*Pyrgus malvae malvae* Linnaeus,1758	2
Lepidoptera	Hesperiidae	*Parnara guttata* (Bremer & Grey,1853)	6
Lepidoptera	Lycaenidae	*Everes argiades* Palls	3
Lepidoptera	Lycaenidae	*Lycaena phlaeas chinensis* (C.Felder,1862)	2
Lepidoptera	Lycaenidae	*Maculinea teleius sinalcon* Murayama	2
Lepidoptera	Lycaenidae	*Acytolepis puspa gisca* Fruhstorfer,1910	3
Lepidoptera	Lycaenidae	*Bothrinia nebulosa* Leech,1890	2
Lepidoptera	Nymphalidae	*Seokia pratti* Leech	2
Lepidoptera	Nymphalidae	*Argyronome ruslana* Motschulsky,1866	3
Lepidoptera	Nymphalidae	*Coenonympha amaryllis* Cramer,1782	1
Lepidoptera	Nymphalidae	*Ypthima conjuncta* Leech,1891	1
Lepidoptera	Nymphalidae	*Argynnis anadyomene* Felder 1861	2
Lepidoptera	Nymphalidae	*Argynnis sagana* Doubleday,1847	1
Lepidoptera	Nymphalidae	*Argynnis laodice* (Pallas)	6
Lepidoptera	Nymphalidae	*Neptis sappho* Pallas,1771	66
Lepidoptera	Sphingidae	*Hemaris affinis* Bremer,1861	1
Neuroptera	Myrmeleontidae	*Pseudoformicaleo nubecula* Gerstaecker 1885	1

Neuroptera	Chrysopidae	*Chrysoperla nippoensis* Okamoto,1914	21
Dermaptera	Labiduridae	*Labidura japonica* De Haan	16
Odonata	Aeshnidae Rambur	*Gastrogomphus abdominalis* McLachlan,1884	6
Mantodea	Mantidae	*Mantis religiosa* Linnaeus	9
Thysanoptera	Thripidae	*Thrips tabaci* Lindeman	39
